# The embryonic blood-cerebrospinal fluid barrier function before the formation of the fetal choroid plexus: role in cerebrospinal fluid formation and homeostasis

**DOI:** 10.3325/cmj.2014.55.306

**Published:** 2014-08

**Authors:** David Bueno, Maryam Parvas, Jordi Garcia-Fernàndez

**Affiliations:** Department of Genetics, Faculty of Biology, University of Barcelona, Barcelona, Catalonia, Spain

## Abstract

Cerebrospinal fluid (CSF) has attracted interest as an active signaling milieu that regulates brain development, homeostasis, and course disease. CSF is a nutrient-rich fluid, which also contains growth factors and signaling molecules that regulate multiple cell functions in the central nervous system (CNS). CSF constitution is controlled tightly and constituent concentrations are maintained narrow, depending on developmental stage. From fetal stages to adult life, CSF is produced mainly by the choroid plexus. The development and functional activities of the choroid plexus, and other blood-brain barrier systems in adults, have been extensively analyzed. However, the study of CSF production and homeostasis in embryos from the closure of the anterior neuropore, when the brain cavities become physiologically sealed, to the formation of the functional fetal choroid plexus has been largely neglected. This developmental stage is characterized by tightly controlled morphological and cellular events in the anterior part of the CNS, such as rapid brain anlagen growth and initiation of primary neurogenesis in the neural progenitor cells lining the cavities, events which are driven by specific molecules contained within the embryonic CSF. In this article, we review the existing literature on formation and function of the temporary embryonic blood-CSF barrier, from closure of the anterior neuropore to the formation of functional fetal choroid plexuses, with regard to crucial roles that embryonic CSF plays in neural development.

The central nervous system (CNS) is one of the most intriguing systems in vertebrates as it controls many crucial processes in living beings, including complex behavior, and its deterioration underlies a myriad of severe and largely incurable diseases. Brain formation begins early on during development, when a portion of the dorsal ectoderm develops into neural ectoderm, forming the neural plate. The neural plate then folds longitudinally to form the neural tube, a rudiment of the CNS. The brain develops out of its anterior portion and the spinal cord out of its posterior portion (1-3) (Figure 1). The architecture of the brain primordium also reveals the existence of connected internal cavities, the cephalic vesicles, which in fetuses and adults become the ventricular system of the brain. During embryonic and fetal development, as well as during adult life, these brain cavities and ventricles are filled with a complex protein-rich fluid, the cerebrospinal fluid (CSF).

**Figure 1:**
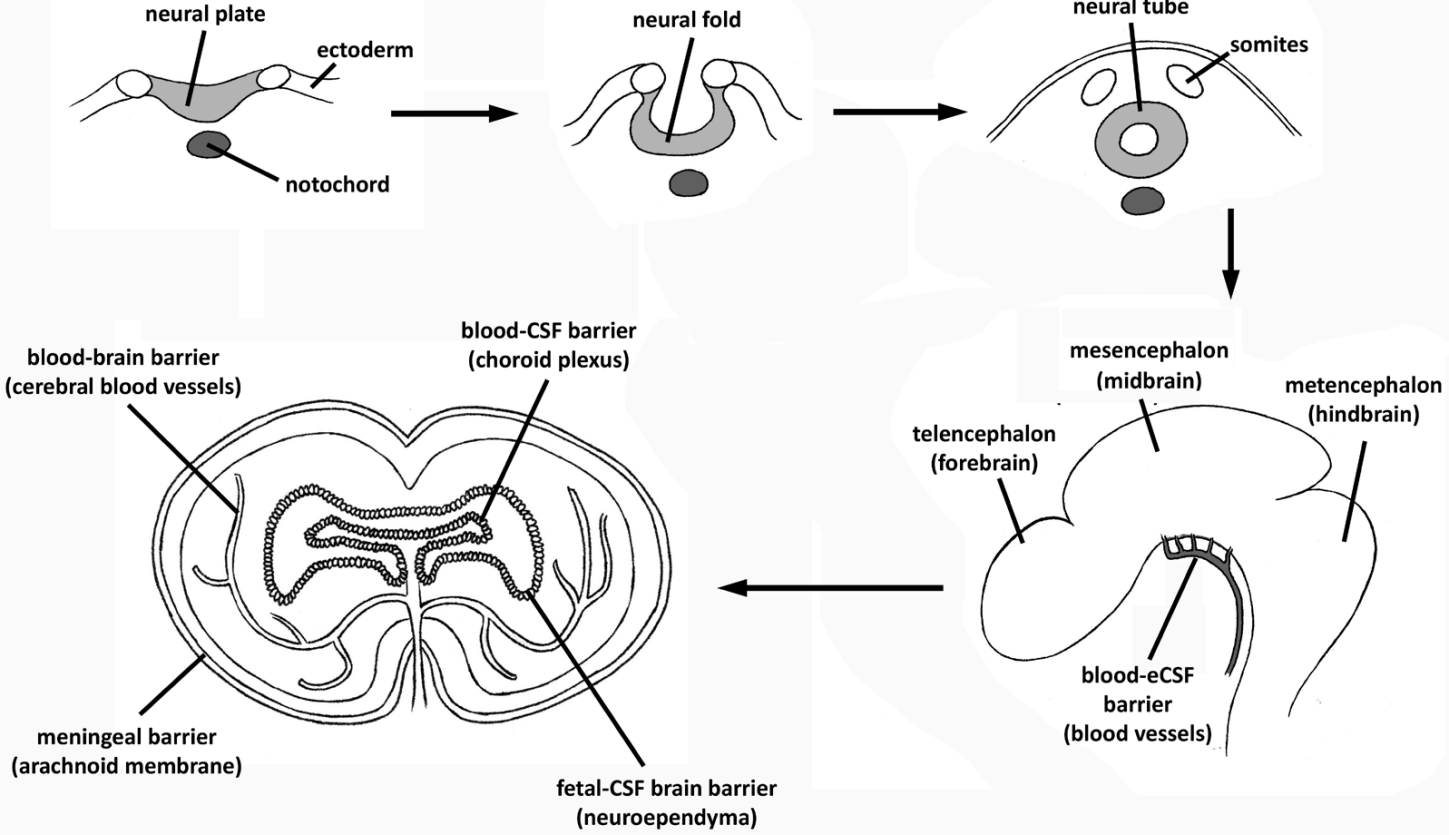
General central nervous system (CNS) development and brain cavity formation. Main barriers are also shown. General CNS development and brain cavity formation is similar in all vertebrates, although these drawings are closer to mammal and avian system.

CSF has intrigued philosophers, physicians, and scientists for the last 4000 years. The earliest mention of a fluid within the brain dates back to ancient Egypt (4). First studies of barriers in the brain are attributed to Herophilus (335-280 BCE), who described the choroid plexus, a highly vascularized secretory epithelium involved in CSF production. Similarly, the first reports on CSF functions are attributed to Galen of Pergamon (129-200/216), who described an alchemical transformation occurring at the base of the brain as a “vital spirit” coming from the blood vessels. A generally accepted hypothesis of CSF physiology is that CSF is primarily produced by the choroid plexus from fetal stages through to adult life. It is a nutrient-rich fluid containing a number of growth factors and other signaling molecules, which bathes the brain and the spinal cord and fills the ventricles of the brain and spinal canal. The study of CSF production and homeostatic control in embryos from the closure of the anterior neuropore to the formation of the choroid plexus has been largely neglected, although this developmental stage plays an important role in brain formation. It is characterized by rapid brain anlagen growth and initiation of primary neurogenesis in neural progenitor cells lining the cavities.

In this article, we review the existing literature on the formation and function of embryonic blood-CSF barriers, from the closure of the anterior neuropore to the formation of choroid plexuses, and we emphasize their importance regarding the roles that the embryonic CSF (eCSF) plays in neural development.

## THE PUZZLING QUESTION OF BARRIER MATURITY

Despite recent efforts to summarize all available information in several comprehensive reviews encompassing fetal stages through to adulthood (4-11), the subject remains somewhat tangled and controversial, especially with respect to brain barrier formation and function during early embryonic development. This is partially due to a common incorrect belief that barriers in the developing brain are immature (4). Another factor contributing to this is the inconsistent terminology used in the literature, which defines early embryonic structures as leaky, tightening, or developing, without considering the actual function they serve (7). Thus, according to the traditional concept there is no reason why the embryonic brain should require an environment of very great chemical constancy for regulated barrier mechanisms to exist. This misconception is amplified by the general term blood-brain barrier (BBB), which is commonly used to describe all anatomical barriers of the brain, with no clear indication as to which particular barrier is discussed.

A further contributing factor is the way in which blood-CSF barrier permeability, usually expressed as the ratio of the concentration of particular molecules in the CSF with respect to blood plasma, has traditionally been interpreted. For small molecules, this ratio is much higher in the developing brain than in the adult brain (11-14), and this has been misinterpreted as evidence of greater barrier permeability (15-18). However, tight junctions, the morphological basis of these barriers, are present from the earliest stages of development between endothelial cells of blood vessels in BBB areas (19), as well as in epithelial cells of the choroid plexuses in the blood-CSF barrier (20,21). To reconcile these two sets of evidence, it has been suggested that different transcellular mechanisms for protein and small molecule transfer operate across the embryonic blood-CSF interface (22).

In the past 15 years, a number of studies reported on the crucial roles of the eCSF (6,10) before the fetal choroid plexus becomes functional. Several proteomic analyses results (9) have suggested that eCSF protein composition in these early stages of development is tightly regulated and dynamic, and it has also been demonstrated that major eCSF protein fractions are produced or stored outside the cephalic vesicles (23), and thus must be transported inside of them, somehow crossing the surrounding neuroepithelium, since at this developmental stage they are physiologically sealed. All these data contradict the general notion that the embryonic brain does not require a controlled environment.

There are three major independent barriers in the adult brain, which gradually start developing early on in fetal stages: 1) the blood-CSF barrier, at the choroid plexus epithelial cells; 2) the blood-brain barrier itself at the endothelium of cerebral blood vessels, and; 3) the arachnoid barrier between the CSF in the subarachnoid space and the dura mater and overlying tissues (Figure 1). The circumventricular organs also form another kind of barrier, allowing linkage between the CNS and peripheral blood flow while protecting the CNS from toxic substances. A number of studies were aimed at investigating the development and functional properties of these barriers (7,24), and found that the choroid plexus is active from fetal stages of development.

Limited information is available on the existence, role, and properties of blood-eCSF barriers in embryos at earlier developmental stages. It is known that embryos possess a temporary barrier between the CSF and brain parenchyma, which only functions as a barrier during the early stages of brain development (25), but very few researchers have investigated the means by which molecules present in the eCSF are transported to this fluid (26). How this fluid is produced and how its homeostasis is controlled, as well as the full range of its functions, continues to be an area of intense research, with important implications for human health (5).

## ARGUMENTS FOR THE PHYSIOLOGICAL NEED FOR A BLOOD-eCSF BARRIER FUNCTION

Through its specific molecules the eCSF plays several crucial roles during early stages of brain development just after the closure of the anterior neuropore, ie, during initial brain anlagen growth and initiation of primary neurogenesis in the neural progenitors of the brain primordium. To explain the need for barriers controlling eCSF composition and homeostasis before the formation of a functional fetal choroid plexus, we will first briefly summarize the functions and composition of this embryonic fluid. In studies where several experimental approaches in chick and mouse embryos were used, it was found that eCSF exerted positive pressure against neuroepithelial walls, generating an expansive force and consequently contributing to the expansion of the brain (27,28). It was suggested that the molecules that mediate this function were chondroitin sulfate proteoglycans and other proteoglycans contained within the eCSF, because of their special osmotic properties (29,30). They are also major components of the extracellular matrix in embryo brain cavities and have a role in facilitating water retention inside the cavities and thereby generate and regulate the inner cephalic hydrostatic pressure.

A study using *in vitro* cultures of mesencephalic neuroectodermal explants from chick embryos at HH24 (corresponding to embryonic day E4) demonstrated that diffusible molecules contained within eCSF actively contributed to the regulation of survival, proliferation, and neurogenesis of neuroepithelial progenitor cells (31). Similarly, it was also demonstrated that diffusible molecules contained within the eCSF actively contributed to the regulation of expression of particular mesencephalic genes, together with the well-known mesencephalic-telencephalic isthmus organizer (32).

Proteomic studies have demonstrated that the eCSF proteome consists of dozens of extracellular matrix proteins, osmotic pressure regulators, ion carriers, hormone-binding proteins, lipid metabolism regulators, and various enzymes and their regulators (33-38), and that it shares many similarities across species during brain development. Conventional proteomic techniques (2D-electrophoresis, in-gel digestion and Electrospray Ionization Mass Spectrometry- analysis) have been used to identify and analyze the eCSF proteome from chick embryos at developmental stage E4, corresponding to maximum neuroepithelial progenitor cell proliferation period and the beginning of neural differentiation (36). Twenty-six proteins contained within eCSF were recognized, identified, and classified into eight groups according to their functional characteristics: 1) gene products related to extracellular matrix proteins, 2) proteins associated with regulation of osmotic pressure and ion transport, 3) proteins related to cell quiescence and death, 4) lipid transport or metabolism proteins, 5) retinol and vitamin D carriers, 6) antioxidant and antimicrobial proteins, 7) intra-cellular proteins, and 8) unknown proteins.

The mammalian eCSF proteome was analyzed in several organisms, including rats and humans. Using the same methods as in chick eCSF proteome analysis (35), thirty-one proteins were identified in rat eCSF and analyzed at an equivalent developmental stage (E12.7, initiation of primary neurogenesis in the brain anlagen). As with the chick eCSF proteome, these proteins included extra-cellular matrix proteins, gene products associated with the regulation of osmotic pressure and ion transport, lipid metabolism regulators, retinol and corticosteroid carriers, as well as antioxidant and antimicrobial proteins. However, rat eCSF also contains a group of proteins that have no functional homologues in chick eCSF, ie, enzymes and enzyme regulators. Moreover, it exhibits an increased number of members of the apolipoprotein family. An extensive proteome analysis of rat eCSF from three different stages, ie, E12.5, E14.5 (taken from both lateral ventricle and 4th ventricle) and E17.5, identified 423, 318, 249, and 382 proteins, respectively. All proteins were again classified into several groups according to their function: extracellular matrix proteins; regulatory molecules, mainly protease inhibitors; cell adhesion proteins; nucleic acid binding proteins; transfer/carrier proteins; immune/defense proteins; receptors; and enzymes (37).

Most of the identified proteins have known physiological functions during embryonic development that are consistent with the overall reported roles for eCSF during CNS development. Functional analyses, both *in vivo* and *in vitro,* of the particular role of some of these gene products at the beginning of primary neurogenesis, before the formation of the choroid plexuses, have revealed their specific roles in the overall function of eCSF in neuroepithelial progenitor cell behavior. For example, it has been reported that the deglycosylation of eCSF proteoglycans with beta-d-xyloside alters brain enlargement in chick embryos (29), and that the immunoblocking of the FGF2 contained within eCSF disrupts neuroepithelial stem cell proliferation and differentiation (39). Similarly, it has also been reported that the low-density lipoprotein lipid fraction, which is transported by the apolipoprotein B contained within eCSF, is also involved in neuroepithelial progenitor cell proliferation and differentiation (40).

Other research has shown that retinol-binding protein (RBP) transports and transfers all-trans retinol from the embryonic plasma to the eCSF, from where it reaches the neuroepithelium and is transformed into retinoic acid, the actual morphogen, by retinoic acid-synthesizing enzymes expressed in the neuroepithelium (41,42). This transport from the blood plasma to the eCSF has also been demonstrated for other molecules, such as FGF2. Intravascularly injected FITC-conjugated FGF2 passes into the eCSF in a regulated manner, whereas control proteins normally not present in the eCSF of similar size and also conjugated to FITC do not have the capacity – or are not allowed – to cross from the blood plasma to the eCSF. In fact, it has also been demonstrated that major eCSF protein fractions are transported from plasma to cephalic vesicles (23) in a developmental stage in which the brain cavities are physiologically sealed due to the neuroectoderm morphology, which includes specific tight junctions. Taken together, all these data suggest a need for a physiological blood-eCSF barrier that controls the initial composition and homeostasis of eCSF, before the formation of functional fetal choroid plexuses. Most probably this blood-eCSF barrier should be physiologically and morphologically different from those of adults and older fetuses, as it may serve specific functions in embryos and must be adapted to developing morphological features.

## MORPHOLOGICAL AND PHYSIOLOGICAL CHARACTERISTICS OF THE INITIAL TEMPORARY BLOOD-eCSF BARRIER

In adults, there are multiple loci of the blood-CSF barrier that control the internal environment of the brain, as both the stability and specificity of this environment are essential for normal brain development and function (43). They significantly impede the passage of virtually all molecules from the blood to the CSF, except for small and lipophilic ones. However, some sets of small and large hydrophilic molecules, such as proteins, can enter the CSF by active transport. Some of these transport systems are known to be receptor-mediated, eg, by the transferrin receptor (44) and there is evidence that other growth factors and cytokines have limited ability to cross blood-CSF barriers (24). Likewise, there are specific transmembrane transporting molecules, present in relatively high concentrations in these endothelial cells, for essential nutrients such as glucose and certain amino acids, vitamins, and ions. Underlying the cellular mechanisms that determine the CSF environment is a fundamental physical barrier at the level of intercellular junctions between cells forming the interface between the blood and the CSF.

On the basis of theoretical considerations, extensive literature, and a growing amount of experimental evidence, it has been argued that during fetal development, the blood-CSF barrier at the level of the developing choroid plexus restricts passage of lipid-insoluble molecules such as gene products by the same mechanism as in the adult, ie, by tight junctions, rendering the paracellular pathway an unlikely route of entry (7). It has also been suggested that proteins are transferred through transcellular routes. Thus, a concept of a functional and dynamic barrier different from that of the adult was introduced, since it is adapted to specific requirements and environment of the early developing nervous system. However, the question still remained of the control of eCSF composition and homeostasis from the closure of the anterior neuropore and before the formation of the functional fetal choroid plexus.

A number of studies published during the last 6 years conducted primarily on chick embryos from E3 to E5 but also in some cases on rat embryos at an equivalent developmental stage (from E12.7 to E13.7) have demonstrated the existence of a functional and dynamic barrier formed by endothelial cells of specific blood vessels as well as by columnar cells of the differentiation neuroepithelium, which acts from the closure of the anterior neuropore, at the beginning of brain primary neurogenesis. This barrier temporarily restricts the passage of lipid-insoluble molecules from the blood to the eCSF and vice versa, thus contributing to eCSF formation and homeostatic control before the choroid plexus becomes functionally active, ie, it is a specific blood-eCSF barrier that contributes to the brain’s internal milieu at this crucial stage of development.

### The impermeability of the cephalic neuroectoderm: a key factor for blood-eCSF barrier activity

One of the key factors why a specific blood-eCSF barrier is needed before the formation of a functional fetal choroid plexus is the impermeability of the cephalic neuroectoderm, which forms an internal, physiologically sealed system of the brain cavities. Early experiments on testing the integrity of this barrier in embryos and fetuses used injected dyes or enzymes, usually trypan blue, Evans blue, or horse-radish peroxidise (HRP), paralleling experiments performed on adults. The injection of dyes gave conflicting results, probably due to different amounts of solution injected into fragile and small embryos (45). Other possible explanations are that dyes reversibly bind to plasma proteins, so the usage of too much of it can result in the movement of unbound dye, which has been shown to be able to pass through many barrier cell types; or facilitated transport of the protein-bound dye.

With respect to HRP, it was shown more than 30 years ago that blood-eCSF and blood-fCSF interface permeability for this molecule in chick embryos began to decrease at E12-E14, ie, 8 to 10 days after the initiation of primary neurogenesis, when the fetal choroid plexus was already fully active (46). According to these experiments, younger embryos may have a free diffusion system for protein transfer between these two embryonic compartments. However, it has recently been demonstrated that the transfer of all other proteins examined to date from the blood plasma to the eCSF is regulated by an active barrier function (47). Why HRP, in contrast to all other proteins examined, is transferred from the blood plasma to the eCSF, has not been yet satisfactorily explained, but it could be related to the presence in chick eCSF of other enzymes with peroxidase activity, which are apparently transported across this barrier (36,47).

Another set of recent findings has also demonstrated that the neuroectoderm of chick embryos is physiologically sealed to small molecules from E4, suggesting the presence of some kind of blood-eCSF barrier controlling their transport. The microinjection of a small-sized tracer, biotin dextran amine of 3 kDa (BDA3000), into the cephalic cavity, or conversely into the outflow of the heart of embryos at E3 (HH20) and E4 (HH23), showed that at E3 this tracer was transferred between the eCSF and the blood plasma and vice versa throughout neuroepithelial cells of the brain cavities, but that from E4 this transport was restricted to a small subset of endothelial and adjacent neuroepithelial cells. This subset of cells is located in the ventral mesencephalon and in the most anterior part of the ventral prosencephalon, lateral to the floor plate (47) (Figure 1). Moreover, histological analysis has shown that in chick embryos at E4, BDA3000 is transported following transcellular routes, which accounts for a physiologically sealed system and suggests the existence of endothelial and neuroepithelial cells specialized in the transport of molecules from the blood plasma to the eCSF and vice versa. The existence of these specialized cells is a key factor for blood-eCSF barrier activity. This situation parallels the functioning of the fetal choroid plexus, as demonstrated by mounting evidence (14,22,48,49).

### Transport of proteins from the blood plasma to the eCSF and vice versa: a crucial factor for eCSF stability

It has been reported that from E3, eCSF has a complex protein composition that differs from that of blood plasma, and that the relative concentration of these proteins varies during development and with respect to adult CSF (50-54). Initially, eCSF derives from trapped amniotic fluid, but after the closure of the anterior neuropore, the brain cavities become a physiologically sealed system, allowing tight regulation of its composition. Thus, at least shortly after the closure of the anterior neuropore, some of the eCSF components may be attributed to encapsulated amniotic fluid, although to date no specific studies have been made regarding their putative influence of brain development.

It has also been demonstrated that at E4, chick eCSF proteome includes molecules whose role during the development of systems other than the eCSF may account for general functions of this fluid, as described above (36,39). Interestingly, most of the molecules identified in chick eCSF are not produced by the neuroectoderm itself, but by other embryonic structures, or alternatively they are stored in the yolk or the white of the egg and taken up by the chorioallantoic membrane (23,39), suggesting that they are transported from the production or storage site to the eCSF, probably via the blood plasma.

Various experiments have demonstrated that the transport of proteins from the blood plasma to the eCSF and vice versa in chick embryos at E4 and E5 is tightly regulated, as is homeostatic control, which is crucial for eCSF function in brain development (47,55). These experiments included the quantification of endogenous chick proteins present in both the blood plasma and the eCSF (ovalbumin, retinol-binding protein, fibroblast growth factor No. 2, and immunoglobulin IgY), as well as the microinjection of several proteins of different molecular size into the brain cavities or alternatively into the outflow of the heart of chick embryos at E4. In the microinjection experiments, the proteins used included both endogenous chick proteins normally present in the eCSF or conversely not normally detected within this fluid, and proteins from sources other than chicks (bovine serum albumin, myosin heavy chain from rabbit, fibroblast growth factor No. 2, plasma retinol-binding protein, a recombinant protein from glutathione-S transferase – from *Schistosoma japonicum*, alcohol dehydrogenase – from *Drosophila lebanonensis,* and ovalbumin).

These experiments demonstrated that the physiological ratio of chick endogenous proteins between the eCSF and the blood plasma was regulated developmentally, as they varied from E3 to E5 in a regulated manner according to their activity in brain development (47). For example, the eCSF/blood plasma ratio for retinol-binding protein is much higher at E4 than at E3 or E5, coinciding precisely with its reported activity in retinol transport from the blood plasma to the eCSF (41), while for fibroblast growth factor No. 2, the eCSF/blood plasma ratio is higher at E5 than at E3 or E4, also coinciding with the developmental period in which this growth factor acts from the eCSF to influence neuroepithelial progenitor cell proliferation (39).

These sets of experiments also demonstrated that the transfer of both chick endogenous proteins and microinjected proteins across the blood-eCSF interface was highly protein-specific (47). For example, chick IgY has never been observed to be transported from the blood plasma to the eCSF, even when its concentration within the blood plasma was experimentally raised by microinjection. Similarly, none of the proteins from sources other than chick, as for example BSA or the recombinant protein glutathione-S transferase/alcohol dehydrogenase, were transported from the blood plasma to the eCSF when experimentally microinjected into the outflow of the heart (with the exception of HRP). Conversely, when chick proteins that under normal physiological conditions are transported between these two embryonic compartments, as for example ovalbumin, fibroblast growth factor No. 2, and retinol-binding protein, were experimentally microinjected into the outflow of the heart – properly linked to FITC to allow their identification with respect to the endogenously produced equivalent proteins – they were actively transported across the blood-eCSF interface (47).

Interestingly, these experiments also demonstrated that the ratio of transfer for these proteins was tightly regulated. In other words, eCSF homeostasis is strongly controlled to maintain fluid stability (47). For example, the transportation from the blood plasma to the eCSF of the above-mentioned proteins linked to FITC did not involve any increase in their concentration within the brain cavities, which remained stable. Moreover, the experimental microinjection of these chick endogenous proteins into the cephalic cavities to directly raise their concentration within the eCSF was rapidly compensated for by the eCSF-blood plasma transport system, which eliminated the excess of proteins in the eCSF within minutes, restoring their normal concentration. Similarly, when the microinjected proteins came from sources other than chick, they were rapidly eliminated from the eCSF. All these data strongly suggest the existence of a blood-eCSF barrier activity before the formation of the functional fetal choroid plexus, acting in both directions to control eCSF formation and homeostasis. In addition, it has also been demonstrated that this precise regulation of protein transport and eCSF homeostasis ensures maximum efficiency of eCSF activity during neural development (55).

A histological analysis of embryos when transporting endogenous proteins linked to FITC or alternatively proteins from sources other than chick demonstrated that within the analyzed developmental period their transfer from the blood plasma to the eCSF and vice versa occurred only at a specific embryo area located in the brain stem lateral to the floor plate, in the ventral mesencephalon and the most anterior part of the ventral prosencephalon (Figure 1). This location does not coincide with the area were the fetal choroid plexus starts to develop, from an invagination of the dorsal roof plate along the midline of the neural tube, indicating that this transient embryonic blood-eCSF barrier function is independent of the choroid plexus, and suggesting that it fulfills the function temporarily lacking from the formation of physiological sealed brain cavities to the initiation of choroid plexus activity. Moreover, histological analysis also showed that this blood-eCSF barrier function includes endothelial cells of specific perineural blood vessels as well as some vascular sprouts located within the neuroectoderm cells and adjacent neuroepithelial cells, using transcellular routes (47).

Immunohistological analysis has revealed the presence of caveolin 1 in endothelial cells of blood vessels involved in the blood-eCSF barrier function as well as in the adjacent neuroectodermal cells (56). Caveolin 1, a member of the caveolin family of proteins, is the main structural component of caveolae, which are 50- to 100-nm vesicular invaginations of the plasma membrane that are involved in transcellular molecular transport, including trancytosis as well as endocytosis, cell adhesion, and signal transduction (57). The presence of caveolin 1 also suggests that these tissues are involved in the transport of molecules from the blood plasma to the eCSF and vice versa, a transport which is highly regulated as shown in different experiments (47). Endothelial cells usually show the highest expression of caveolin 1 (58), which is also found in the human BBB-provided vessels (59). Interestingly, caveolin 1 has also been detected in the same embryonic area in rat embryos at an equivalent developmental stage (E12.7), including parallel blood vessels and their adjacent neuroectodermal cells (56). Although few data are available on protein transport from the blood plasma to the eCSF at this developmental stage in mammals, the presence of caveolin 1 in the very same region involving both endothelial and neuroepithelial cells has led to speculation on the existence of a barrier function in mammals acting in the same way as demonstrated in chick embryos (56).

### Water, ion, and glucose transport: from eCSF formation to function in brain development

During early CNS development, the increase in brain cavity volume is accompanied by a parallel increase in eCSF volume (6), rendering the control of water flux and regulation of solute transport critical. Rapid membrane water transport is mediated by a family of transmembrane proteins that form molecular water channels, the so-called aquaporins (AQPs), which have been identified in epithelial and endothelial cells at numerous locations in higher vertebrates (60-65). Of the 12 AQPs described to date (AQP0 to AQP11), AQP-1, AQP-4, and AQP-9 play a relevant role in water transfer in the adult brain (64), and AQ1 and AQ4 also play a role in water transport across brain barriers. AQ1 has been shown to be expressed at very early stages of choroid plexus development in a range of mammalian species, including humans (65), and it has been reported that AQ4 is present in adult brain endothelial cells (66), although this is still under debate. Findings from several studies suggest that choroid plexus apical expression of AQP-1 is closely related to the rate of CSF formation. For example, AQP-1 null mice have lowered CSF formation and pressure (67).

Similarly, ion transfer appears to be critical for eCSF production and brain anlagen growth. Ions can move through cell membranes both actively by ion pumps and passively via ion channels. In the adult, the ionic composition of the brain environment is kept very stable as this is essential for normal nerve conduction. Fluctuations in blood ionic levels are not reflected in fluctuations within the CNS and there are clear ion gradients between blood and the brain (43). Also in adults, AQP4 co-localizes with the inwardly rectifying K+ channel Kir4.1 (68), and it has been suggested that together, these two proteins play a key role in K+ and water balance. However, very few data are available on developing embryos. For example, the presence of a Cl^−^ and Mg++ ionic gradient between CSF and brain has been reported in sheep embryos at E44–50 (term is 150 days), indicating the presence of an effective ion pump mechanism (69).

A study using immunohistochemical procedures and polymerase chain reaction (PCR) analysis revealed that AQP1, AQP4, and Kir4.1 were present in chick and rat embryos in the very same blood vessels where specific protein transport was also detected (70). In chick embryos, AQP1 is present from E4, and AQP4 and Kir4.1 from E5. Interestingly, these specific transporters are also detected in rat embryos at equivalent developmental stages; AQP1 is detected from E12.7 and AQP4 and Kir4.1 from E13.7. These data confirm that a blood-eCSF barrier function controls eCSF composition and homeostasis from the early stages of brain development in chick embryos, including water and ion influx, thus regulating E-CSF osmolarity, and it has been proposed that a similar blood-eCSF barrier is also present in mammals at equivalent brain development stages (56,70), although no data on protein transport are available in these organisms at these early stages of brain development.

Apart from water and ion influx, transport of molecules as energy sources for the developing neuroepithelium is also of high importance. Glucose is an important energy source for the brain, although to a lesser extent in the fetus than in the adult (71). Glucose is transported across cell membranes mainly by the GLUT family of solute carriers, including GLUT1, which in adults is located in the brain barriers. It has also been reported that choroid plexus epithelial cells also express GLUT1, starting shortly after they have differentiated (24). Similarly, a study using immunohistochemical procedures and PCR analysis has demonstrated that in chick and rat embryos, GLUT1 is also present in the very same blood vessels where protein transport and the presence of AQP1, AQP4, and Kir4.1 have been detected (70), supporting the roles ascribed to the blood-eCSF barrier in early brain development with respect to eCSF formation and homeostasis.

## CONCLUDING REMARKS

### The importance of the blood-eCSF barrier for controlling eCSF composition and homeostasis in early brain development

The ionic stability, neurochemical environment, and normal functioning of the brain are crucially dependent on the integrity of its barrier systems in adults as well as during development. The importance of the blood-eCSF barrier in the control of eCSF composition and homeostasis, and thus, indirectly, in early brain development, has also been tested by disrupting it with chemical procedures (72). Chick embryos were treated with 6-aminonicotinamide gliotoxin (6-AN), a known antimetabolite of nicotinamide that blocks protein transport across the BBB (73) by halting transcellular caveolae transport, and it was found that the lack of protein transport across the blood-eCSF barrier clearly influences neuroepithelial cell survival, proliferation, and neurogenesis. This blockage also disrupts water influx to the eCSF, altering brain anlagen growth.

The roles ascribed to the early and temporary blood-eCSF barrier functions reviewed in this paper confirm the crucial importance of the eCSF milieu for the developing brain at the initiation of primary neurogenesis, after the closure of the anterior neuropore and before the fetal choroid plexus starts to be fully functional. They also strongly suggest that this transient barrier function should be considered as an actual barrier system, operating in early embryos at a specific location and comprising both endothelial cells of particular blood vessels and the adjacent neuroectodermal cells. This barrier system, which operates transcellularly, includes specific transporters for particular molecules, such as aquaporins, as well as transporters for ions and glucose. The highly specific protein transport by the caveolae system also suggests the presence of particular transporters for proteins, in order to discriminate among proteins and also to detect their concentration within the eCSF, as the homeostasis of this fluid is tightly regulated.
